# Are There Critical Fatigue Thresholds? Aggregated vs. Individual Data

**DOI:** 10.3389/fphys.2016.00376

**Published:** 2016-08-31

**Authors:** Daria Neyroud, Bengt Kayser, Nicolas Place

**Affiliations:** ^1^Institute of Sport Sciences, University of LausanneLausanne, Switzerland; ^2^Department of Physiology, Faculty of Biology and Medicine, University of LausanneLausanne, Switzerland

**Keywords:** task failure, endurance performance, peak twitch, maximal voluntary contraction, critical threshold, neuromuscular fatigue

## Abstract

The mechanisms underlying task failure from fatiguing physical efforts have been the focus of many studies without reaching consensus. An attractive but debated model explains effort termination with a critical peripheral fatigue threshold. Upon reaching this threshold, feedback from sensory afferents would trigger task disengagement from open-ended tasks or a reduction of exercise intensity of closed-ended tasks. Alternatively, the extant literature also appears compatible with a more global critical threshold of loss of maximal voluntary contraction force. Indeed, maximal voluntary contraction force loss from fatiguing exercise realized at a given intensity appears rather consistent between different studies. However, when looking at individual data, the similar maximal force losses observed between different tasks performed at similar intensities might just be an “artifact” of data aggregation. It would then seem possible that such a difference observed between individual and aggregated data also applies to other models previously proposed to explain task failure from fatiguing physical efforts. We therefore suggest that one should be cautious when trying to infer models that try to explain individual behavior from aggregated data.

The typical answer when asking someone who just terminated a fatiguing task why stopped is “I just couldn't go on any further”. If this suggests a conscious but forced decision to de-recruit the activated motor units for the task at hand, the underlying mechanisms forcing someone to terminate a fatiguing task are still not understood (Kayser, 2003). Given the great variety in physical tasks and their differing physiological constraints, it is unlikely that a single mechanism applies, just as it is likely that various types of effort will share some common pathways leading to the disengagement from the task at hand. For example, during dynamic exercise such as running or cycling, the cardiovascular strain is much greater than during an isometric contraction of a specific muscle group (Sidhu et al., 2013). For the former the sensation of effort is multimodal (e.g., breathlessness, palpitations, leg pain), while for the latter it is essentially related to the muscular effort *per se* (e.g., pain, loss of force) as the cardiorespiratory strain is limited.

The various mechanisms potentially implicated in exercise termination have been the focus of a great number of studies (for reviews see Kayser, 2003; Hunter et al., 2004; Marcora and Staiano, 2010; Enoka et al., 2011; Noakes, 2012; Amann et al., 2013; Enoka and Duchateau, 2016; Fan and Kayser, 2016; Taylor et al., 2016). In particular the role of peripheral fatigue, i.e., involving mechanisms located beyond the motor endplate, has been researched extensively. Functionally, peripheral fatigue can be evaluated by quantifying reductions in evoked forces induced by single or paired supramaximal electrical or magnetic stimulations delivered over a motor nerve trunk before and after a fatiguing exercise. If peripheral fatigue extent has been proposed as a key determinant causing task failure by some authors (Amann and Dempsey, 2008; Amann et al., 2009, 2013; Amann, 2011; Sidhu et al., 2014; Blain et al., 2016), its role in the termination of different types of muscular effort is still hotly debated (Marcora, 2009; Marcora and Staiano, 2010; Johnson et al., 2015; Morales-Alamo et al., 2015).

## A critical peripheral fatigue threshold as a determinant of task failure?

A recently postulated mechanism shared between various types of muscular effort is a so-called “critical peripheral fatigue threshold,” proposed by Amann et al. (2006, 2009, 2013) and Blain et al. (2016). According to those authors, peripheral fatigue normally does not exceed a certain individual critical threshold. If, during a given task, this threshold is reached, individuals either terminate it (open-ended tasks) or reduce the intensity (closed-ended tasks) (Amann et al., 2009). This critical peripheral fatigue threshold concept emerged from the observation of consistently reproducible peak twitch force reductions immediately following various cycling bouts to task failure (open-end) (Amann et al., 2006, 2007, 2009, 2011; Romer et al., 2007; Amann and Dempsey, 2008), as well as 5-km cycling time trials (closed-end) (Amann et al., 2006; Blain et al., 2016). Further support for such a critical peripheral fatigue threshold hypothesis was that at task failure of these open and closed-ended tasks, a greater reduction in peak twitch was reached following selective blockade of sensory afferents with intrathecal fentanyl injection compared to saline (Amann et al., 2009, 2011; Blain et al., 2016). Given the direct evidence of III-IV afferents involvement in exercise regulation from animal studies (Darques and Jammes, 1997; Dousset et al., 2004), this suggests that sensory type III-IV afferents might play a critical role in the regulation of a tightly regulated individual “permissible” extent of peripheral fatigue.

Yet, recent findings by Morales-Alamo et al. (2015) are difficult to reconcile with this notion of a critical fatigue threshold. In their hallmark study, the subjects performed 10-s long all-out isokinetic sprints before, and 10 or 60 s after an incremental maximal cycling test. Immediately at task failure of the incremental exercise, a bilateral cuff was placed around both thighs and inflated to occlude leg blood flow and hence prevent metabolite clearance. Given the (measured) low levels of phosphocreatine (PCr) and increased levels of adenosine diphosphate (ADP) at task failure, mitochondrial respiration was still high when the cuffs were inflated and the authors calculated that the little oxygen remaining was depleted within the following 3 s. Despite acidosis, anaerobic glycolysis and metabolites continued to accumulate during the ischemic recovery, as highlighted by the higher muscle lactate concentration at 60 s of occlusion compared to at 10 s. The ischemic recovery thus induced a greater metabolic disturbance and, as such, it should be expected that type III and IV afferent firing was at the very least maintained if not increased during this period (Jankowski et al., 2013; Laurin et al., 2015). As the cuff was deflated only immediately before the beginning of the sprints performed either 10 or 60 s after the incremental maximal test, a poorer sprint performance was expected after the 60 s recovery period compared to the 10 s one. Surprisingly, not only was the power developed after 10 s higher than the maximum power reached at task failure of the preceding incremental exercise test, the sprint after 60 s of ischemic recovery reached higher power than the one after 10 s. These findings suggest that despite a *milieu interne* expected to strongly stimulate type III-IV afferents, motor drive was not inhibited as much during the 10 s all-out sprints as compared to at task failure of the incremental test. Even though evoked force loss was not directly quantified in this study, nor were the experiments repeated after intrathecal fentanyl injection, the results nevertheless question a universal critical peripheral threshold hypothesis, at least for 10-s long all-out sprints.

Further evidence that questions the critical peripheral threshold comes from the different extents of evoked peak twitch reductions found at task failure from a cycling bout performed at 80% of peak power in different studies. Goodall et al. (2012) reported an average reduction of 20%, Sidhu et al. (2014) of 46%, and Amann et al. (2011) of 34%, even though all three studies used an identical exercise task. One possible explanation for the lesser peak twitch decrease found at task failure by Goodall et al. (2012) than by Amann et al. (2011) could be that task failure can occur before reaching a critical peripheral fatigue. This possibility is supported by recent results obtained by Johnson et al. (2015), who reported that the extent of evoked force loss at task failure of a cycling bout was less when realized after prior upper body exercise. It is further possible that such differences between studies reporting reductions in evoked force might be explained by differences in the characteristics of the participants involved (e.g., different training status and other inter-individual variability). On the other hand, supposing that different participant cohorts were involved in each of their studies, it is striking how the different studies conducted by Amann and colleagues consistently found a reproducible peak twitch force loss. Nevertheless, given the above mentioned discrepancies, it would seem that the critical peripheral fatigue threshold hypothesis based on “interindividual consistency of the degree of end-exercise fatigue” (Broxterman et al., 2015) needs further testing.

To foster the debate we here review a series of recent studies in which losses in MVC and evoked forces as well as changes in voluntary activation level (VAL) induced by a fatiguing effort were quantified (Table 1). Studies were included if they involved exercise tasks where the subjects were asked to continue for as long as possible, i.e., until task failure, and quantified MVC and evoked force losses. The tasks varied from isometric contractions with various muscle groups to dynamic exercise tasks such as cycling at a fixed power output until task failure. There clearly are discrepancies in the extent of evoked force losses following a given exercise (see Table 1). However, attention should be paid to the time point at which evoked forces were evaluated in these studies, as the delay between the moment of task failure and the subsequent evaluation affects the extent of the latter because of recovery (Neyroud et al., 2012; Froyd et al., 2013). As highlighted in Table 1, at task failure from a 20% MVC sustained isometric contraction of the knee extensors, reductions in evoked forces varied from 3% in Place et al. (2005) to 24% in Matkowski et al. (2011) even though a similar break (~ 20 s) was provided between task failure and the electrically-evoked contraction. Although single and paired stimuli are classically used as indexes of peripheral fatigue, it should be mentioned that the number of stimuli delivered might affect the factors constraining evoked force production (Parmiggiani and Stein, 1981). To sum up, in light of these studies, and given MVCs performed with similar levels of voluntary activation rates, the existence of a critical peripheral fatigue threshold does not appear obvious.

**Table 1:** **Summary of studies that quantified maximal voluntary contraction (MVC), evoked force and voluntary activation level (VAL) changes after various exercise**.

**Study**	**Muscle**	**Fatiguing task**	**Post evaluation at**	**TTF s**	**MVC loss %**	**Evoked force loss %**	**VAL loss %**
Matkowski et al., 2011	KE	20% MVC to TF one leg	not specified	295	−37	−24^a^	−13
Matkowski et al., 2011	KE	20% MVC to TF two legs	not specified	245	−26		−7
Neyroud et al., 2012	KE	20% MVC to TF	at TF	246	−51	−37^a^	−7
Place et al., 2005	KE 35°	20% MVC to TF	20−30 s	974	−28	−3^a^	−19
Place et al., 2005	KE 75°	20% MVC to TF	20−30 s	398	−28	−4^a^	−14
Place et al., 2007	KE	40% MVC to TF	20−30 s	101	−16	−3^a^/−15^b^	−6
Kalmar and Cafarelli, 1999	KE	50% MVC to TF Pla	immediately after TF	66	−30	−55^b^	
Kalmar and Cafarelli, 1999	KE	50% MVC to TF Caf	immediately after TF	82.5	−30	−55^b^	
Neyroud et al., 2013	KE	50% MVC to TF	at TF	77	−34	−28^a^/−36^b^	−5
Amann et al., 2006	KE	cycling at 83% peak power to TF in Nx	2 min 30	489	−9	−24^a^/−34^b^	
Amann et al., 2006	KE	cycling at 83% peak power to TF in Hx	2 min 30	270	−11	−23^a^/−32^b^	
Amann et al., 2006	KE	cycling at 83% peak power to TF in hyperoxia	2 min 30	1162	−9	−24^a^/−32^b^	
Amann et al., 2011	KE	cycling at 80% peak power to TF	3 min	522	−10	−34^b^	−1
Amann and Dempsey, 2008	KE	cycling at 83% peak power to TF	4 min	~600	−10	−36^b^	0
Goodall et al., 2012	KE	cycling at 80% peak power to TF in Nx	2 min 30	486	−17	−19^b^	−9
Goodall et al., 2012	KE	cycling at 80% peak power to TF in Hx	2 min 30	216	−25	−30^b^	−18
Sidhu et al., 2014	KE	cycling at 80% peak power to TF	~40s	588	−16	−46^b^	−10
							
Rupp et al., 2015	KE	40% MVC to TF in Nx	immediately after TF	458	−18	−10^a^/−18^b^	−9
Rupp et al., 2015	KE	40% MVC to TF in Hx	immediately after TF	449	−16	−6^a^/−16^b^	−12
Neyroud et al., 2013	PF	50% MVC to TF	at TF	220	−30	−7^a^/−1^b^	−13
							
Yoon et al., 2007	EF	20% MVC to TF men	immediately after TF	636	−17	−23^b^	−10
Yoon et al., 2007	EF	20% MVC to TF women	immediately after TF	1020	−32	−33^b^	−17
Yoon et al., 2008	EF	20% MVC to TF young	immediately after TF	864	−27	−28^b^	−14
Yoon et al., 2008	EF	20% MVC to TF old	immediately after TF	1770	−38	−33^b^	−13
Neyroud et al., 2013	EF	50% MVC to TF	at TF	72	−40	−59^a^/−72^b^	−6
Yoon et al., 2007	EF	80% MVC to TF men	immediately after TF	25	−16	−37^b^	−4
Yoon et al., 2007	EF	80% MVC to TF women	immediately after TF	24	−15	−29^b^	−6
Yoon et al., 2008	EF	80% MVC to TF young	immediately after TF	24	−15	−33^b^	−4
Yoon et al., 2008	EF	80% MVC to TF old	immediately after TF	32	−9	−18^b^	−2
							
Fuglevand et al., 1993	FDI	20% MVC to TF	~30 s	534	−40	−55^a^	
Fuglevand et al., 1993	FDI	35% MVC to TF	~30 s	246	−30	−54^a^	
Fuglevand et al., 1993	FDI	65% MVC to TF	~30 s	66	−19	−10^a^	
							
Neyroud et al., 2013	ADD	50% MVC to TF	at TF	114	−37	−60^a^/−63^b^	−2

TTF, time to task failure; TF, task failure; Nx, normoxia; Hx, hypoxia; KE, knee extensors; PF, plantar flexors; ADD, adductor pollicis; EF, elbow flexors and FDI, first dorsal interosseous.

a Indicates that peripheral fatigue extent was measured by evoking a 100-Hz paired stimulation whereas

b means that it was measured by evoking a single stimulation.

## Could a more global critical threshold be involved in task failure?

Alternatively, the extant literature on neuromuscular fatigue appears compatible with the existence of a more global critical threshold based on MVC force loss. Indeed, in view of the different studies reported in Table 1, it appears that at failure of tasks realized at a given intensity, MVC force losses were similar. A MVC force loss of ~30–40% seems to be consistently observed following sustained isometric contractions performed with the knee extensors at 20% MVC when the MVCs were realized a few seconds after task failure (Place et al., 2005; Matkowski et al., 2011). Similar MVC force losses were found when a similar task was performed with the elbow flexors (Yoon et al., 2007, 2008). This suggests that MVC force loss might be tightly regulated to not exceed a certain threshold.

Similarly to what was observed for evoked forces, the time at which MVC force loss is assessed affects its extent (Neyroud et al., 2012; Froyd et al., 2013). Yet, if results from studies evaluating MVC force loss at a similar time delay after task failure are compared, then its extent appears to be rather consistent. When sustained isometric contractions are performed, MVC force losses also appeared consistent between the various studies for a given intensity (c.f. Table 1). Interestingly, a similar MVC force loss was found following a 50% MVC sustained isometric contraction performed to task failure with four different muscle groups, whereas reductions in evoked forces differed (Neyroud et al., 2013). Again, these results would support a tight regulation of MVC force loss. Accordingly, it can be hypothesized that instead of being limited by the extent of peripheral fatigue (reflected by reductions in evoked forces), exercise termination might result from a certain degree of MVC force loss being reached, the latter being exercise-intensity dependent.

**Figure 1:**
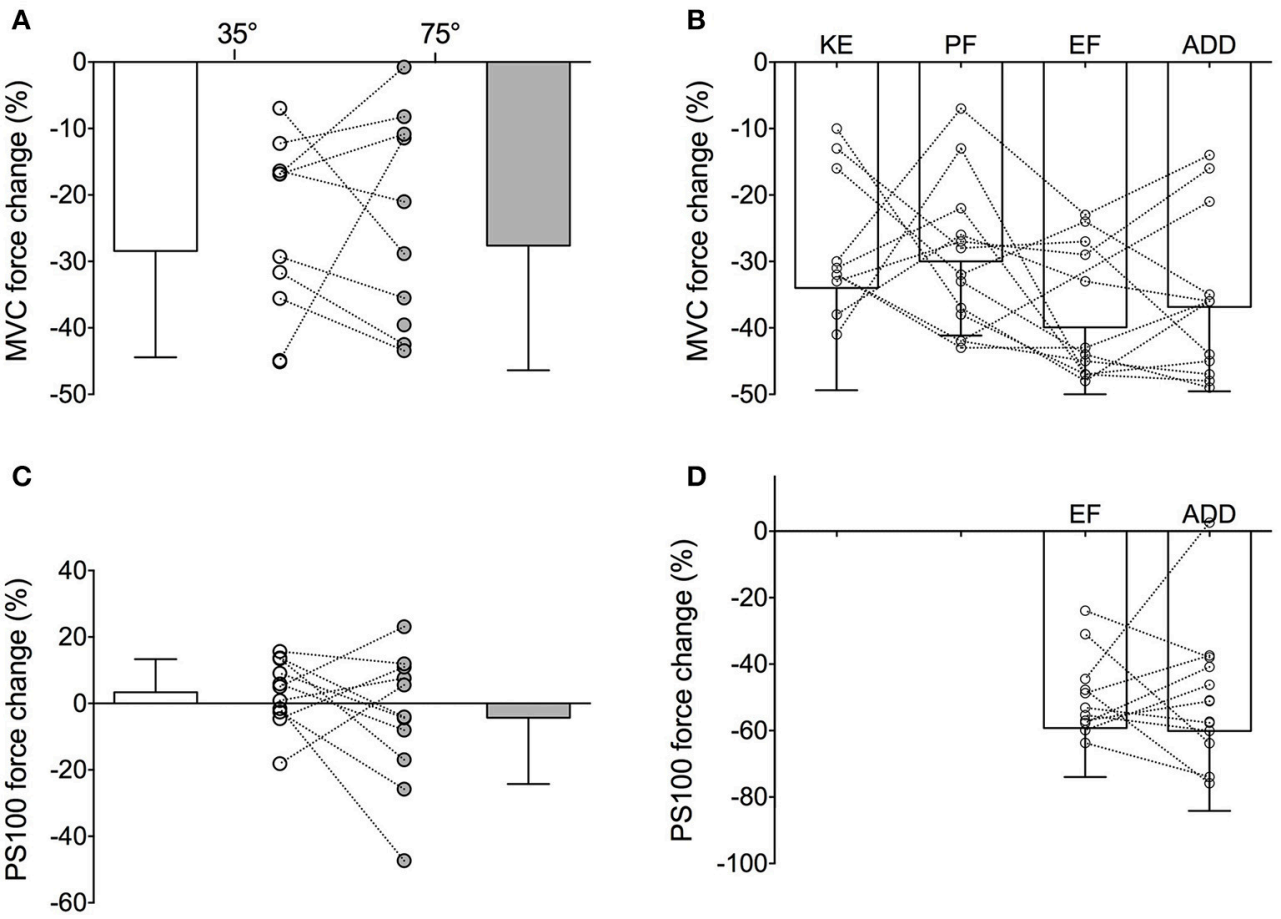
**Maximal voluntary contraction (MVC) force and 100-Hz evoked (PS100) force changes at task failure of (i) (A,C) a 20% MVC sustained isometric contraction performed with the knee extensors at knee angles of 35° (unfilled) and 75° (filled) of knee flexion and (ii) (B,D) a 50% MVC sustained isometric contraction performed with four muscle groups**. For the illustration of the present purpose, PS100 force loss was only represented for the elbow flexor and *adductor pollicis* tasks in panel D as peripheral fatigue extent differed in the two other muscle groups. KE, knee extensors; PF, plantar flexors; EF, elbow flexors and ADD, *adductor pollicis*.

However, although this new hypothesis appears seductive, close examination of individual data shows that such a critical global threshold based on MVC force loss does not hold at the individual level and rather seems a mere artifact of data aggregation. For example, considering the data obtained by Place et al. (2005) following a sustained isometric contraction at 20% MVC to task failure performed with the knee extensors at two different knee angles (35° vs. 75° of knee flexion), it appeared that averaged MVC force losses were similar between the two tasks (−28 ± 16 and −28 ± 19% following the 35° and 75° task, respectively). Yet, when considering individual values, it can be seen that MVC force loss was greater following the 35° task than the 75° one in four of the nine participants, whereas the opposite was found in the five other participants (Figure 1A). It thus appears that the similar averaged MVC force loss observed in this study between the two different tasks was a mere reflection of half of the participants showing one behavior and the other half behaving in the opposite manner. A similar finding is observed from the results obtained in a study comparing MVC force loss induced by a sustained isometric contraction at 50% MVC to task failure performed with four different muscle groups (Neyroud et al., 2013, Figure 1B).

The similar reduction in evoked forces reported between the two fatiguing tasks performed in Place et al. (2005) can also be ascribed to cancelation of two opposite behaviors shown by the participants (some showing a greater evoked force reduction after the 35° task whereas evoked forces were reduced to a greater in extent in some others after the 75° task, Figure 1C). Similarly, in Neyroud et al. (2013), the evoked force loss measured at failure of the exercise involving elbow flexors and the *adductor pollicis* was similar, whereas a look at the individual values (Figure 1D) clearly showed that half the participants displayed greater reductions following the elbow flexor task and the other half following the *adductor pollicis* task.

As a critical threshold (global or peripheral) would be physiologically relevant only at the individual level, the above mentioned observations highlight the importance of considering interpretation of individual data and not only of group means, despite statistics. Indeed, when group means are compared and models/theories inferred from them (such as done when results of several studies are put together and new interpretations are inferred), caution should be taken to avoid drawing wrong conclusions by making ecological errors [i.e., deducing inferences about individual data from group averages (Sheppard, 2003)]. Models aiming to explain task failure and exercise performance should therefore be inferred from individual data and not averaged ones. However, for that to be possible, future studies should consider presenting both mean and individual data as proposed in Figure 1. Adopting such a manner of presenting results might lead to better comprehension of the mechanisms regulating exercise performance and responsible for task failure as both individual and mean data would be available to the reader.

## Author contributions

All authors listed, have made substantial, direct and intellectual contribution to the work, and approved it for publication.

### Conflict of interest statement

The authors declare that the research was conducted in the absence of any commercial or financial relationships that could be construed as a potential conflict of interest.
